# Hematopoietic Id Deletion Triggers Endomyocardial Fibrotic and Vascular Defects in the Adult Heart

**DOI:** 10.1038/s41598-017-03160-7

**Published:** 2017-06-08

**Authors:** Corey Chang, Qingshi Zhao, J. Patrick Gonzalez, Jung H. Kim, Kamal Alzahrani, Dominic Del Re, Diego Fraidenraich

**Affiliations:** Department of Cell Biology and Molecular Medicine, New Jersey Medical School, Rutgers Biomedical and Health Sciences, 185 South Orange Avenue/Medical Science Building G-624, Newark, NJ 07103-2501 United States of America

## Abstract

Inhibitor of DNA binding (Id) proteins play important roles in regulating cardiac development via paracrine signaling. Id1/Id3 knockout mice die at mid-gestation with multiple cardiac defects. Single Id knockout studies have not reported cardiomyopathies. To bypass embryonic lethality we used Tie2CRE-mediated recombination to conditionally delete Id1 against global Id3 ablation (Id cDKOs), which develops adult-onset dilated cardiomyopathy. We confirm upregulation of thrombospondin-1 (TSP1) in Id cDKO hearts. Colocalization studies reveal increased TSP1 expression in the vicinity of endothelial cells and near regions of endocardial fibrosis/disruption. Downstream fibrotic molecules were upregulated. Endocardial capillary density was reduced with evidence of vascular distention. Treatment of Id cDKO cardiac explants with LSKL, a peptide antagonist of TSP1 activation of TGFβ, reversed the increased expression of fibrotic molecules. We conducted bone marrow transplant experiments in which we transferred bone marrow cells from Id cDKO mice into lethally irradiated WT mice. The majority of WT recipients of Id cDKO bone marrow cells phenocopied Id cDKO cardiac fibrosis 4 months post-transplantation. Injection of LSKL into adult Id cDKO mice led to downregulation of fibrotic molecules. The results prompt caution when bone marrow transfers from individuals potentially carrying mutations in the Id axis are applied in clinical settings.

## Introduction

The inhibitor of DNA binding genes (Id1-4) are dominant negative antagonists of basic-helix-loop-helix (bHLH) transcription factors known to orchestrate cardiac development in the embryo as demonstrated by the observation that Id1/Id3 double knockout (Id DKO) embryos die at midgestation, exhibiting multiple cardiac defects (e.g., ventricular septal defects, trabecular meshwork disruption and a characteristically thin myocardial wall) reminiscent of the ‘thin myocardial wall syndrome’^1, 2^. Within the embryonic heart, Id genes are specifically expressed in nonmyocardial layers including the epicardium, endocardium, endothelium and endocardial cushion^3^. The expression of Id genes outside of affected tissues (e.g., myocardium) suggests that Id may exert its effects through paracrine signaling mechanisms. Intraperitoneal injection of IGF1 (a downstream epicardial id dependent factor) in mothers harboring Id DKO embryos rescued these pups to birth^3^. However, these pups died at birth and histological characterization of their hearts reveal that although the caliber of the myocardium was restored, multiple cardiac defects persisted most of which resided in the innermost regions of the heart. This observation led us to hypothesize that endocardial and endothelial Id signaling plays an important role in cardiac development. The embryonic lethality of Id DKOs limits our ability to the study of the role of Id genes in the heart postnatally.

To bypass this limitation, eliminate Id compensation and investigate the role of Id genes in a tissue layer specific manner, we crossed Id3 KO mice with mice harboring flox mutations around the Id1 gene (Id1 Flox) and targeted Id ablation to the endocardium and endothelium by utilizing the Tie2Cre driver, thereby generating Id conditional double knockout Tie2Cre^+^Id1^F/F^id3^−/−^ or Tie2Cre^+^Id1^F/−^Id3^−/−^ mice (Id cDKO)^4^. These mice developed into adulthood with multiple novel phenotypes including anemia/splenomegaly, dilated cardiomyopathy and wound healing defects^4, 5^. We previously reported that adult Id cDKOs develop a cardiac phenotype by 6 months of age characterized by endocardial disruption, endomyocardial fibrosis, increased perivascular fibrosis, hypertrophic changes and impaired cardiac function (decreased ejection fraction and fractional shortening)^4^. Microarray analysis of 5–6 month old Id cDKO hearts revealed dysregulation of angiogenic, fibrotic and hypertrophic markers^4^.

## Methods

### Genotyping and Mouse Colonies

Mice harboring flox insertions flanking the Id1 gene were crossed with mice with null mutations in Id1 and Id3 to generate Id1^F/F^id3^−/−^ and Id1^F/−^Id3^−/−^ (Id control mice). Id control mice were then crossed against Tie2Cre mice through a series of successive breedings to ultimately yield Tie2Cre+Id1^F/F^id3^−/−^ and Tie2Cre+Id1^F/−^Id3^−/−^ mice (Id cDKO). For verification of Cre/LoxP recombination, B6;129S3-Gt(ROSA)26Sor^tm1Sor^/J mice were crossed with breeding intermediates. For bone marrow transplantation studies, GFP transgenic mice, C57BL/6-Tg(UBC-GFP)30Scha/J were crossed with Id control and Tie2Cre intermediates to generate GFP-labeled Id control and Id cDKO mice. All mice used in experiments were congenic and possessed the C57BL/6J genetic background except for Tie2Cre mice, which were backcrossed 8–10 times against a pure C57BL/6J background to achieve >99% background purity. Genotyping PCR was performed using previously established primers and protocols^4, 5^. All animal experiments were approved by the Institutional Animal Care and Use Committee (IACUC) of Rutgers New Jersey Medical School and performed in accordance with relevant guidelines and regulations.

### Cardiac Explant Culture

Freshly isolated mouse hearts were immediately cut into 1mm transverse sections using sterilized cutting blades with a cutting matrix. Sections were rinsed in 1X DPBS and placed in serum starvation medium (low glucose DMEM supplemented with 1 mg/mL BSA with no FBS) for 1 hr. For the serum incubation experiment, sections were incubated in serum starvation medium supplemented with 2% mouse serum isolated from different mice. For experiments using recombinant proteins and peptides, sections were treated with serum starvation medium supplemented with 20 μM LSKL (Anaspec), 125 ng/mL recombinant TSP1 (R&D), 500 ng/mL recombinant IGFbp3 (R&D) and 200 ng/mL TSP1 Ab (Neomarkers). Cardiac explants were incubated at 37 °C for 6 hrs with 5% CO_2_ under rocking conditions. Following treatment, the supernatant was collected and centrifuged at 900 g at 4 °C to isolate the cells in suspension. The resulting cell pellet was washed in 1X PBS, snap frozen in liquid nitrogen and submerged in Trizol reagent (Invitrogen). Cardiac explants were also snap frozen in liquid nitrogen and suspended in Trizol reagent. Trizol reagent was also added directly to the well to isolate RNA from the adhering cells at the bottom of the well. Trizol fractions were combined and subsequently processed to extract total RNA following manufacturer’s protocol.

### Bone Marrow Transplantation

After clearing away muscle tissue, femurs and tibias were rinsed in 70% ethanol followed by 1X PBS wash. Total bone marrow cells were freshly isolated by flushing the femurs and tibias of mice from each study group using a 27G1½ PrecisionGlide needle (Becton-Dickinson) with IMDM (Invitrogen) supplemented with 2% FBS (Invitrogen). Debris was removed by straining the cells through a 40 μM nylon cell strainer (BD Falcon). Cells were centrifuged at 2000 rpm (900 × g) for 10 mins at 4 °C. Cells were subsequently resuspended and counted using a hemacytometer (Bright-Line). 5 × 10^6^ bone marrow cells were resuspended in 100 μL 1× PBS and injected into the retroorbital sinus using a 27G1½ PrecisionGlide needle. Bone marrow injections were performed under isoflurane anesthesia (3% isoflurane in oxygen) under sterile conditions. Prior to bone marrow transplantation, recipient mice were first lethally irradiated with 11 Gray (Gy) of radiation from a Cesium-137 (Cs-137) dual source to ablate the endogenous bone marrow cells. Efficacy of irradiation was assessed by designating one mouse as an irradiation control, which does not receive bone marrow injection. These mice typically die off between 10–20 days post irradiation. Efficiency of reconstitution was checked at varying points post-transplantation by quantifying percentage of GFP positive cells per total number of gated cells. 95–100% donor cell incorporation was deemed full reconstitution. Following transplantation, mice were given autoclaved acidified (pH 2.3) water supplemented with enrofloxacin (Baytril) at a dosage of 600 μL per 1 L of volume for up to one month post-transplantation. In the correction study, Id cDKO recipients were transplanted with WT donor bone marrow cells to create ‘forward transplanted’ or FBMT mice. Conversely, in the dysregulation study, WT recipients were transplanted with GFP donor bone marrow cells to create ‘reverse transplanted’ or RBMT mice.

### LSKL Injection

LSKL (Anaspec) was injected into the retroorbital sinus at a dosage of 4 μg per gram of mouse body weight. Mice were then sacrificed 6 hours after the injection and tissue were acquired for further analysis.

### Statistical Analysis

Data are presented as the mean ± s.e.m. (standard error of the mean). Statistical analysis was performed using the GraphPad Prism 5 Software. Independent t-tests were performed for comparisons between two groups. One-way ANOVA was performed for comparisons across multiple groups. The probability level of p < 0.05 was considered to be statistically significant. All experiments were performed in triplicate.

## Results

### Activation of TSP1/CTGF/collagen in Id cDKO hearts

**Figure 1:**
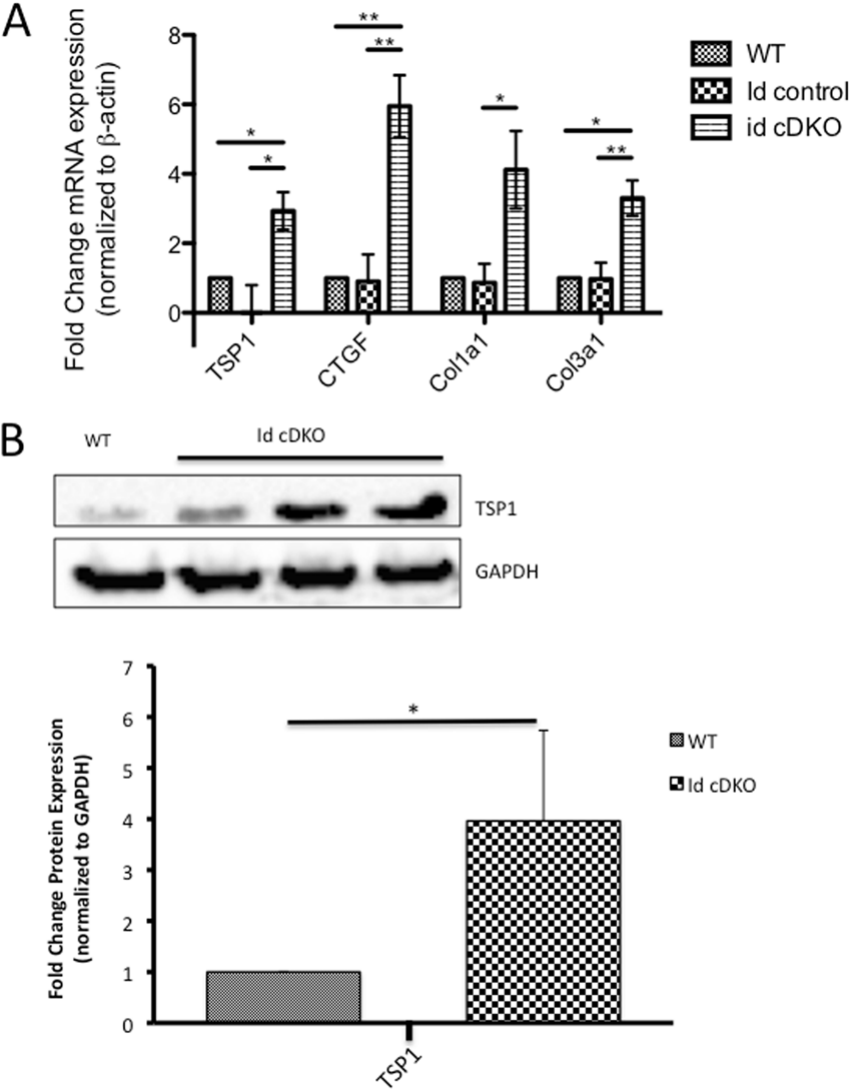
Activation of TSP1/CTGF/collagen in Id cDKO hearts. (**A**) RT PCR analysis of WT (n = 3), Id control (n = 6) and Id cDKO (n = 6) hearts between 5–6 months of age. mRNA expression normalized to β-actin. A one-way ANOVA was used to test for differences in mRNA expression amongst the three groups. Data presented as fold change relative to WT levels. mRNA levels were significantly different across the three group for the following markers: Thrombospondin-1 (TSP1): F (2, 12) = 6.12, p = 0.01; connective tissue growth factor (CTGF): F (2, 12) = 11.05, p = 0.002; collagen I (Col1a1): F (2, 12) = 4.07, p = 0.04; collagen III (Col3a1): F (2, 12) = 6.07, p = 0.02. *p < 0.05; **p < 0.01 (**B**) Representative western blot for TSP1 in 6 month old WT and Id cDKO hearts, and quantification. Protein levels were normalized to GAPDH. Quantitation provided on right panel is expressed as ratios denoted on x-axis. Data presented as fold change relative to WT levels.

Based on microarray studies from prior studies^4^, we identified significant upregulation of thrombospondin-1 (TSP1) in Id cDKO hearts. TSP1 mRNA levels were found to be increased 2.93 ± 0.54-fold (p = 0.01) in Id cDKOs relative to WT hearts (Fig. 1A). No significant differences in TSP1 mRNA expression were observed between Id control and WT hearts while Id cDKO cardiac TSP1 levels were found to be significantly higher than Id control levels (p = 0.01) (Fig. 1A). These trends were confirmed by western blot analysis, demonstrating a 3.9 fold increase in TSP1 protein levels relative to WT levels (Fig. 1B). Connective tissue growth factor (CTGF), a secreted protein found to be strongly upregulated in heart failure^6, 7^ was elevated 5.95 ± 0.89-fold (p = 0.009) relative to WT levels (Fig. 1A). No significant differences in CTGF expression were observed between Id control and WT hearts although a significant difference was found between Id cDKO and Id control hearts (p = 0.002). Collagen I (Col1a1) mRNA levels were observed to be upregulated 4.12 ± 1.12-fold (p = 0.08) relative to WT levels (Fig. 1A). No significant differences were observed between WT and Id control hearts although Id cDKO hearts showed a significant elevation in Col1a1 mRNA levels compared to id control levels (p = 0.03). Collagen III (Col3A1) mRNA levels were also found to be significantly upregulated 3.30 ± 1.12-fold (p = 0.03) in Id cDKO hearts relative to WT hearts (Fig. 1A). No significant difference was detected between WT and Id control hearts although a significant difference was observed between Id cDKO and Id control hearts (p = 0.007). Taken together, these results suggest activation of the TSP1/CTGF/collagen pathway in Id cDKO hearts.

### Defective endothelial network formation and localization of TSP1 upregulation in the vicinity of cardiac endothelial cells within the endocardium

**Figure 2:**
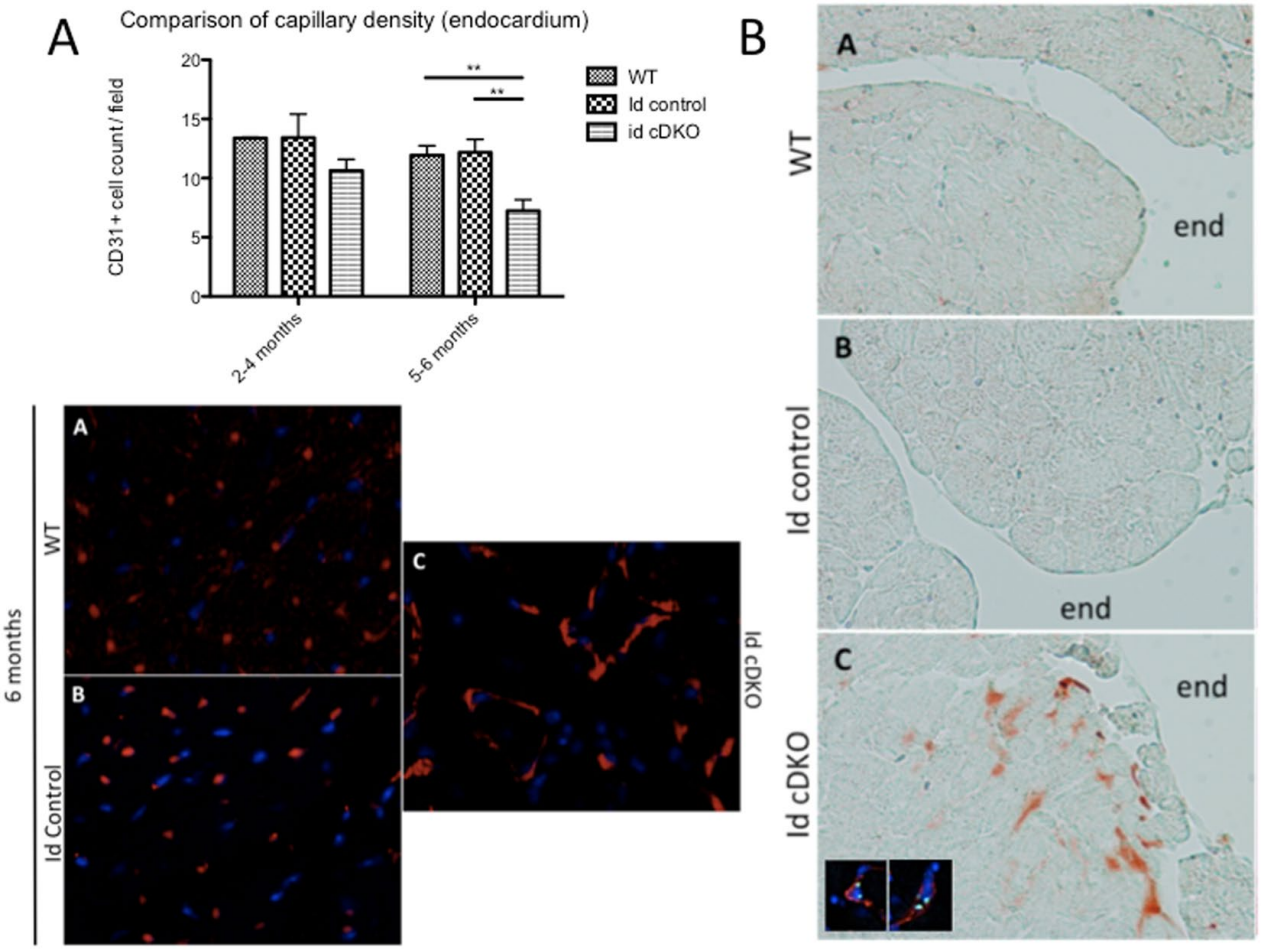
Defective endothelial network formation and localization of TSP1 upregulation in the vicinity of cardiac endothelial cells within the endomyocardium. (**A**) Comparison of capillary density within the endocardium of isolated hearts at 2–4 months and 5–6 months of age. CD31 quantitation within the endocardium of WT (n = 2 at 2–4 months; n = 4 at 5–6 months), Id control (n = 3 at 2–4 months; n = 7 at 5–6 months) and Id cDKO hearts (n = 3 at 2–4 months; n = 7 at 5–6 months). n = number of mice. Data presented as average number of counted CD31+ cells per field (Mean ± SEM). The panel on the bottom demonstrates representative CD31 stain (red) for endothelial cells with DAPI counterstain (blue) for nuclei. Images A–C illustrate representative images for WT, Id control and Id cDKO hearts respectively (Magnification: 200x) *p < 0.05, **p < 0.01. (**B**) Representative TSP1 immunostain of 6 month old Id cDKO heart. Images A–C demonstrate WT, Id control and Id cDKO hearts respectively. Majority of TSP1 immunostain found within the endomyocardium. (Magnification: 200x). end = endocardium. Images (inset) show representative TSP1/CD31 dual immunofluorescence with DAPI counterstain of 6 month-old Id cDKO hearts. TSP1 (green), CD31 (red), DAPI (blue) (Magnification: 400x).

Prior studies demonstrated that TSP1 serves as an effector of the Id pathway and has an angiostatic effect on endothelial cells^8, 9^, Analysis of CD31 immunostained cardiac sections revealed a marked decrease in capillary density within the endocardium that was observed as early as 2 months of age but was significant by 5–6 months of age (p < 0.01) (Fig. 2A). This decrease in capillary density and observed defect in endothelial network is congruent with TSP1’s known angiostatic effect. To determine the pattern of TSP1 expression within the Id cDKO heart, cardiac sections were stained with the anti-TSP1 antibody Ab-4 (clone A6.1). TSP1 immunostains demonstrated a pattern that was interstitial and most heavily localized to the endocardium and endomyocardium (Fig. 2B). In particular, TSP1 staining was observed near regions of endocardial disruption. Confocal microscopy on CD31/TSP1 dual-stained sections of Id cDKO hearts revealed foci of TSP1 in the immediate vicinity of CD31 positive endothelial cells (Fig. 2B). These findings demonstrate that TSP1 expression in Id cDKO hearts is localized to endothelial cells mostly within the endocardial regions.

### Increased apoptosis in 5–6 month old Id cDKO hearts

**Figure 3:**
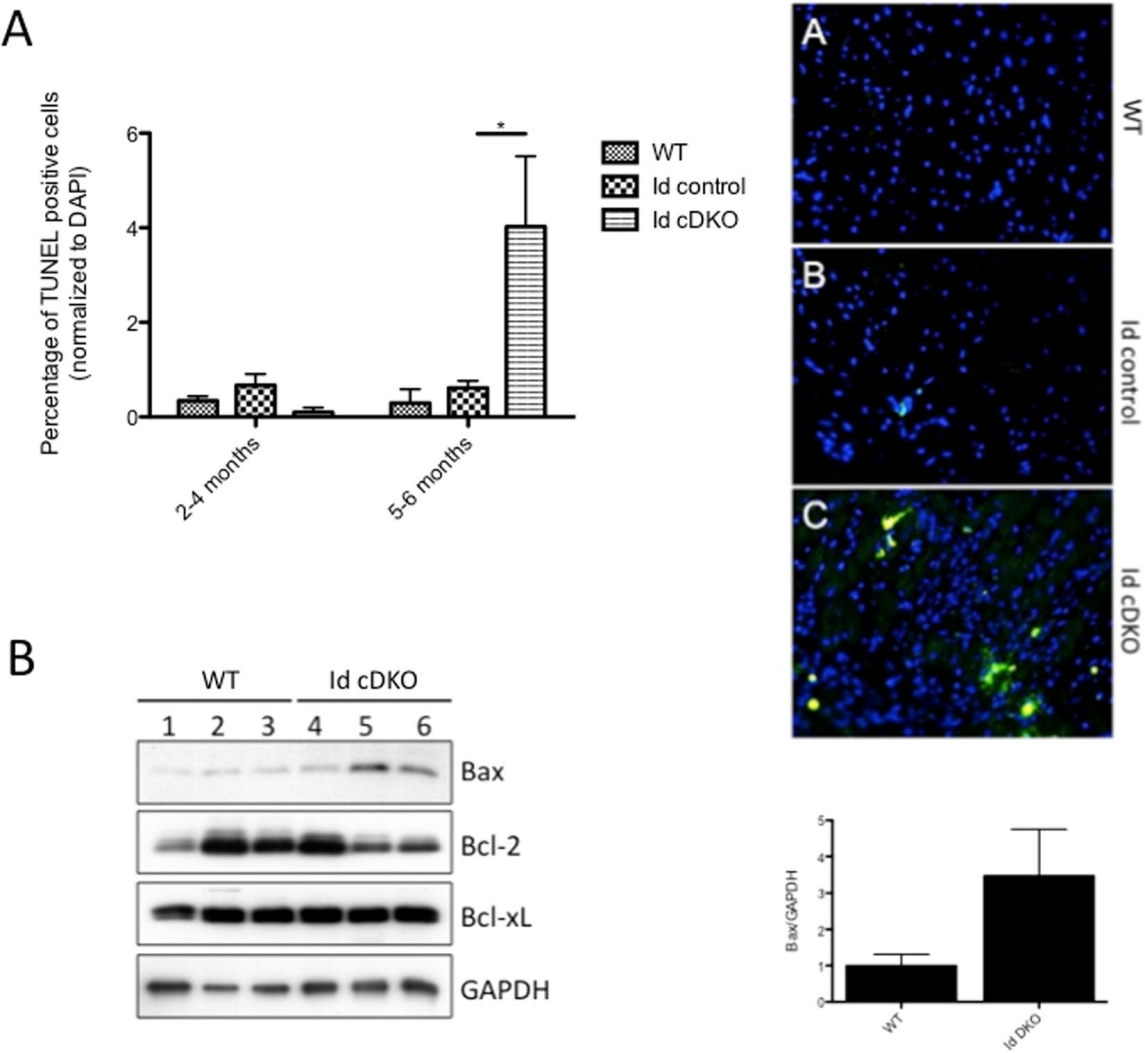
Increased Apoptosis in 5–6 month old Id cDKO hearts. (**A**) Quantitation of TUNEL positive cells in WT (n = 3 at 2–4 months; n = 3 at 5–6 months), Id control (n = 3 at 2–4 months; n = 5 at 5–6 months) and Id cDKO (n = 3 at 2–4 months, n = 4 at 5–6 months) hearts. Data expressed as percentage of TUNEL positive cells normalized to DAPI count. Panel on right demonstrates representative TUNEL (green) stain with DAPI (blue) counterstain. Images A–C demonstrate WT, Id control and Id cDKO hearts respectively. *p < 0.05 (**B**) Representative western blot for Bax, Bcl-2, Bcl-xL and GAPDH in the hearts of 6 month old WT (lanes 1–3) and Id cDKO (lanes 4–6) mice. Panel on right shows quantitation of Bax expressed as fold change relative to WT levels. n = number of mice. Data presented as Mean ± SEM.

Prior studies have shown that TSP1 not only exerts angiostatic effects but also induces apoptosis by activating the downstream caspase death pathway^10, 11^. The upregulation of TSP1 in Id cDKO hearts raises the question of whether TSP1 may be playing an apoptotic role in this system. Quantitative TUNEL analysis revealed that while no significant differences in apoptosis were observed across the three groups (WT, Id control, Id cDKO) at 2–4 months of age, a significant increase in TUNEL positive cells were detected at 5–6 months of age (Fig. 3A). Western blot analysis of Id cDKO hearts revealed an overall increase in Bax protein level (3.8 fold increase compared to WT levels) but no significant changes in Bcl-2 or Bcl-xL levels (Fig. 3B). Taken together, these results suggest that Id cDKO hearts develop evidence of increased apoptosis at later stages of adult life.

### Transfer of Id null bone marrow cells into WT recipients (R-BMT) leads to development of endomyocardial fibrosis

**Figure 4:**
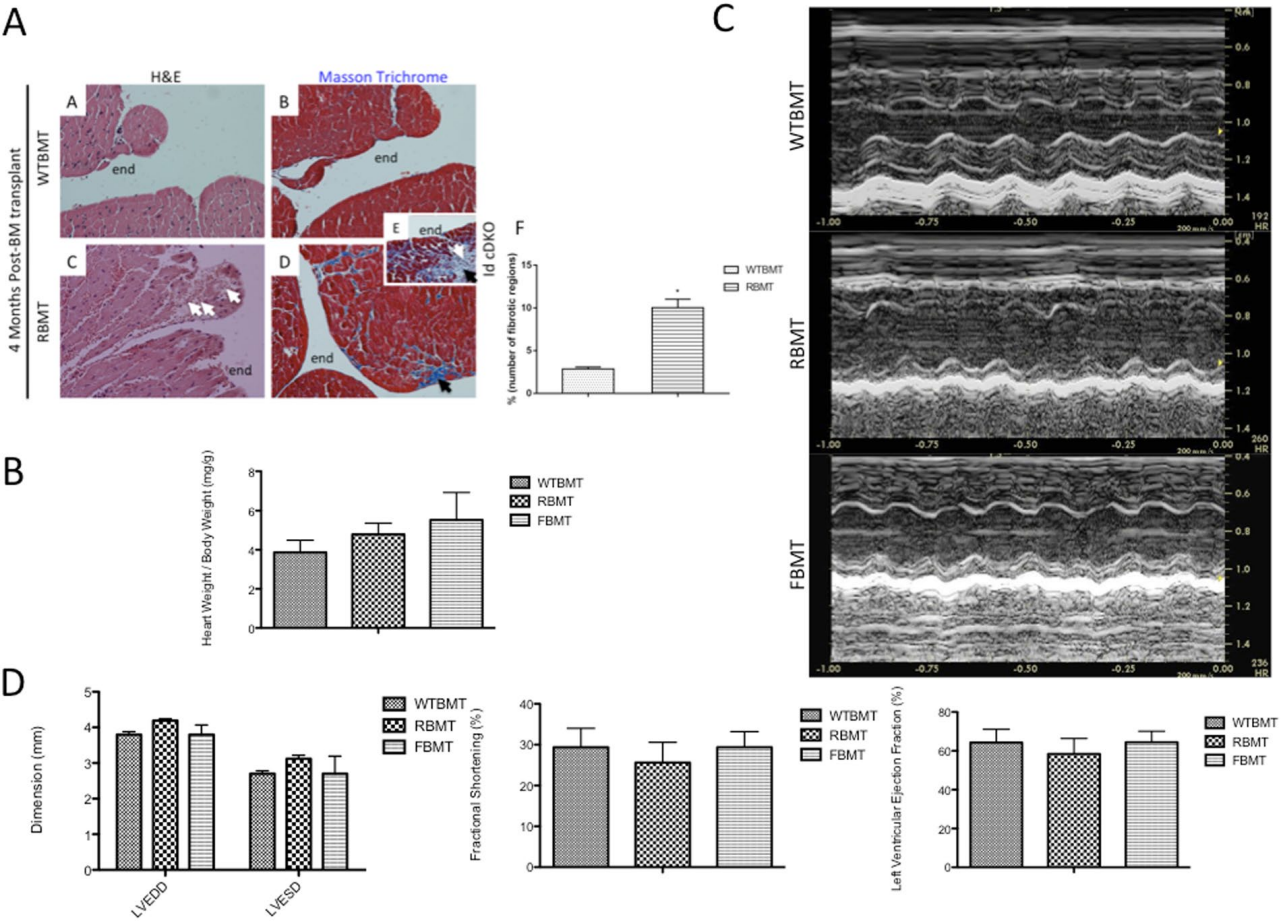
Transfer of Id null bone marrow cells into WT recipients (R-BMT) leads to development of endomyocardial fibrosis. (**A**) Representative H&E stains and Masson Trichrome stains of WTBMT (Images A&B) and RBMT (Images C&D) hearts of recipients 4 months post transplantation. Inset E shows typical Id cDKO cardiac pathology for comparison. White arrows show areas of endocardial disruption and mononuclear invasion. Black arrows show regions of fibrosis. (Magnification: 200x). Bar graph in F shows quantitation of % of number of fibrotic regions (number of regions containing fibrosis)/(number of regions containing cardiac muscle) × 100. WTBMT: n = 2, 10 sections and RBMT: n = 4, 15 sections. *p < 0.05. Data presented as Mean ± SEM. (**B**) Quantitation of heart weight/body weight ratio expressed as mg/g of body weight of WTBMT (n = 11), RBMT (n = 7) and FBMT (n = 5). (**C**) Representative echocardiographs of 6 month-old WTBMT, RBMT and FBMT mice. (**D**) Cardiac function parameters (Dimension, Fractional Shortening and Left Ventricular Ejection Fraction) of 6 month-old WTBMT (n = 14), RBMT (n = 9) and FBMT (n = 5) mice. LVEDD = left ventricular end diastolic diameter, LVESD = left ventricular end systolic diameter. n = number of mice. Data presented as Mean ± SEM.

Because Tie2 is also expressed in hematopoietic cells and a number of hematopoietic defects (e.g., anemia and splenomegaly) were apparent in Id cDKO mice^4^, the question arises as to whether hematopoietic Id contributes to the dilated cardiomyopathy observed in Id cDKO mice. To address this question, a series of bone marrow transplantations were performed to control the Id status of the bone marrow cells. In the forward transplantation setup (FBMT), lethally irradiated Id cDKO recipients were transplanted with WT bone marrow donor cells and subsequently observed for evidence of phenotypic rescue. In the reverse transplantation setup (RBMT), lethally irradiated WT recipients were transplanted with Id cDKO bone marrow donor cells and subsequently observed for evidence of pathology. Full reconstitution occurred. Histological analysis of hearts from WTBMT (wild type recipients of wild type bone marrow) and RBMT mice at 6 months of age (4 months post transplantation) revealed evidence of cardiac fibrosis in the endomyocardium of RBMT hearts reminiscent of the cardiac fibrosis observed in Id cDKO hearts with areas of endocardial disruption and interstitial fibrosis (Fig. 4A). Evidence of cardiac pathology was found in 62% of RBMTs (Suppl. Table 1). Total collagen content in RBMT was not different from that of WTBMT (0.52 ± 0.07 μg hydroxyproline/mg left ventricle compared to 0.63 ± 0.07 μg hydroxyproline/mg left ventricle respectively, p = 0.13). 70% of FBMTs demonstrated evidence of cardiac disease (Suppl. Table 1). Penetrance of pathology does not appear to correlate with disease severity in the donor. Heart weight/body weight ratio showed a non-significant trend towards higher levels in both RBMT and FBMT mice compared to WTBMT controls (Fig. 4B). Parameters of cardiac function were not significantly different in RBMT and FBMT mice compared to WTBMT (Fig. 4C and D).

### Activation of TSP1/CTGF/Collagen in RBMT hearts

**Figure 5:**
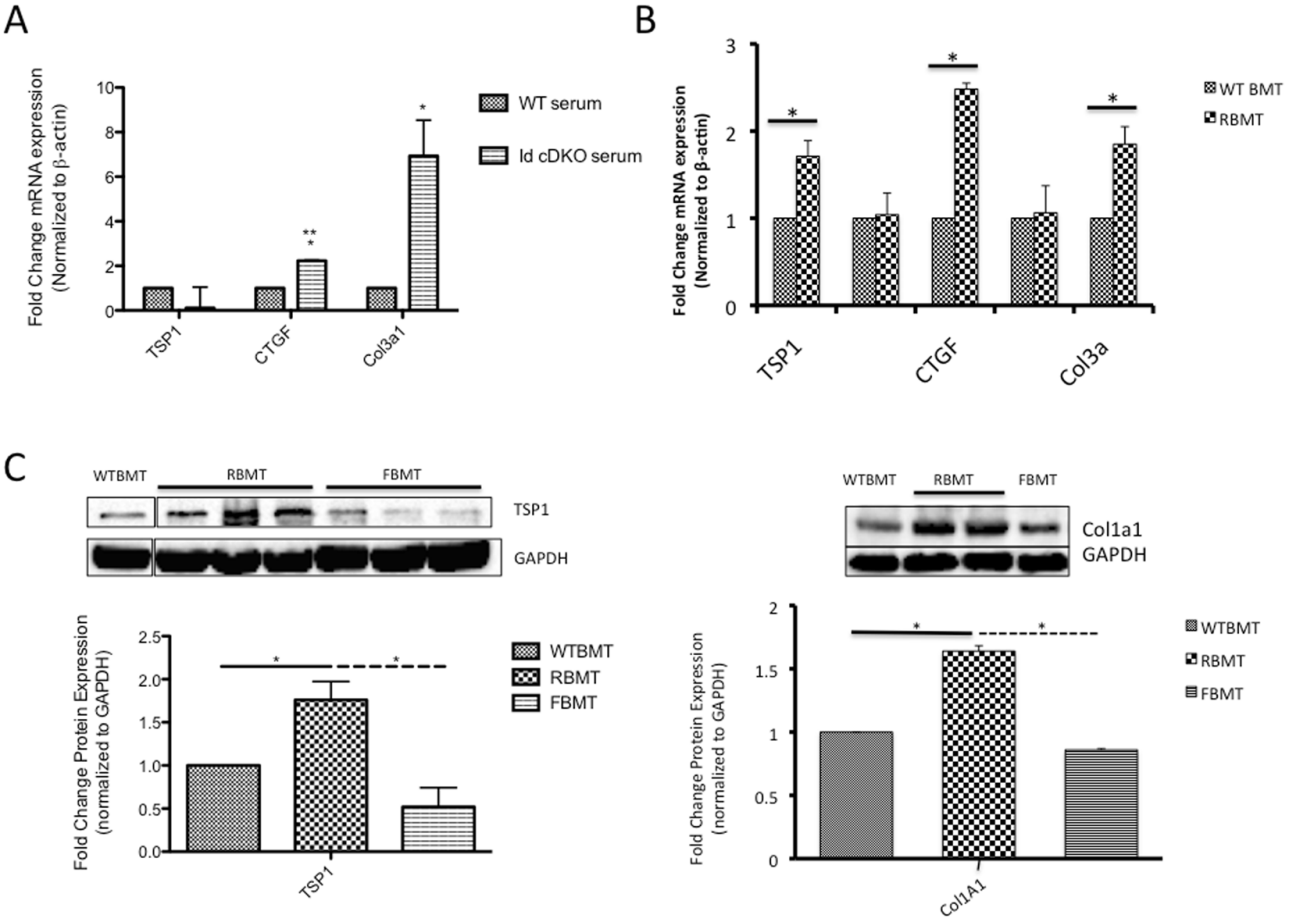
Activation of TSP1/CTGF/Collagen in RBMT hearts. (**A**) Incubation of 1 mm WT cardiac sections (obtained from 2 month-old mice) in medium with 2% mouse serum from 2 month old WT and Id cDKO mice. Data expressed as fold change in mRNA expression levels of TSP1, CTGF and Col3a1 in WT cardiac explants treated with Id cDKO serum relative to WT serum treated controls. WT serum treatment (n = 3), Id cDKO serum treatment (n = 3). mRNA expression normalized to β-actin. *p < 0.05, **p < 0.01. (**B**) Fold change in mRNA expression for TSP1, TGFβ1, CTGF, Col1a1 and Col3a1 in 6 month old WTBMT (n = 4) and RBMT (n = 7) mice. *p < 0.05. (**C**) Representative western blots for TSP1 in WTBMT, RBMT and FBMT hearts at 6 months of age. Lane of WTBMT was not adjacent to lanes of RBMT and blot was cropped. Image of uncropped blot is available as Supplementary Figure 1. Graph on bottom displays quantitation of TSP1 band intensity normalized to GAPDH. *p < 0.05.

The observation that RBMT mice develop cardiac fibrosis reminiscent of the pattern of fibrosis observed in Id cDKO donors leads to the question of whether abnormalities in the circulating factors secreted within the serum by these bone marrow cells can trigger fibrotic pathways in the heart. To test this question, an *ex vivo* cardiac explant experiment was developed in which 1 mm sections of WT hearts were incubated with 2% serum obtained from WT and Id cDKO mice under rocking conditions at 37 °C in 5% CO_2_ for 5 hrs after which point RNA was extracted for real time PCR analysis (Fig. 5A). While no significant differences in TSP1 expression were apparent, CTGF and Col3A1 were upregulated by 2.23 ± 0.01 fold (p < 0.01) and 6.93 ± 0.93 fold (p < 0.05) respectively (Fig. 5A).

The observation of incipient cardiac fibrosis in RBMTs reminiscent of the cardiac fibrosis observed in Id cDKOs leads to the question of whether the same TSP1/CTGF/collagen cascade of molecular events is activated in RBMT hearts. RT PCR analysis of hearts from bone marrow transplanted mice revealed a 1.71 ± 0.18 fold increase in TSP1 expression in RBMT vs. WTBMT levels (p = 0.02) (Fig. 5B). CTGF expression was significantly increased 2.48 ± 0.07 fold (p = 0.05) in RBMT hearts (Fig. 5B). Increased expression of Col3A1 by a fold change of 1.85 ± 0.20 (p = 0.0003) was detected in RBMT hearts (Fig. 5B). No significant changes in Col1A1 were observed. Western blot analysis confirmed 1.76 ± 0.21 fold increase (p = 0.05) in TSP1 protein levels in RBMT hearts relative to WTBMT levels (Fig. 5C). Conversely, western blot analysis revealed a trend towards decreased TSP1 protein levels (0.52 ± 0.22 fold decrease, p = 0.37) in FBMT hearts compared to WTBMT hearts. TSP1 protein levels were significantly increased in RBMT hearts compared to FBMT hearts. Together these findings suggest that TSP1/CTGF/collagen are activated in RBMT hearts.

### Injection of LSKL in Id cDKO mice antagonizes TSP1 expression and leads to downregulation of collagen

**Figure 6:**
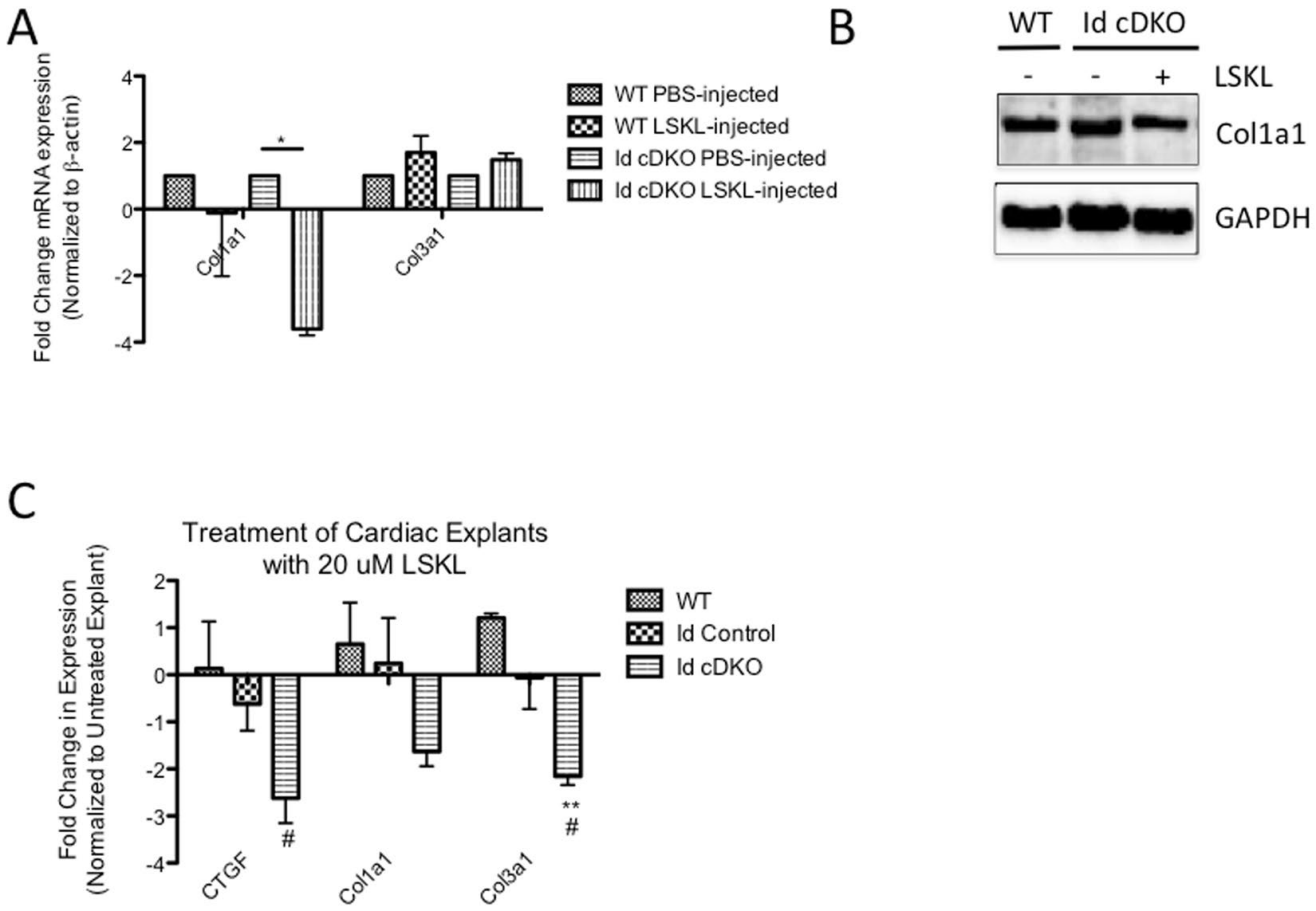
Injection of LSKL in Id cDKO mice antagonizes TSP1activity and leads to downregulation of collagen. (**A**) RT PCR analysis of hearts from WT and Id cDKO injected/not injected with LSKL. Fold change in mRNA expression for Col1a1, Col3a1 in Id cDKO hearts at 2 months of age with PBS vs. LSKL. WT (n = 3 for each treatment group), Id cDKO (n = 3 for each treatment group). *p < 0.05 (**B**) Representative western blot for Col1a1 in hearts from PBS and LSKL injected mice at 2 months age. Quantitation of band intensity provided in graph below, expressed as fold change relative to untreated levels. (**C**) RT PCR analysis of cardiac explants treated with LSKL. Data expressed as fold change in mRNA expression in cardiac explants relative to untreated controls from WT, Id control and Id cDKO mice following treatment with 20 μM LSKL. WT (n = 3 for each treatment gorup), Id control (n = 4 for each treatment group), Id cDKO (n = 3 for each treatment group) *p < 0.05 (Id cDKO vs. WT), **p < 0.01 (Id cDKO vs. WT), ^#^p < 0.05 (Id cDKO vs. id control), ^##^p < 0.01 (Id cDKO vs. Id control).

To determine whether activation of the TSP1/CTGF/collagen pathway can be antagonized at the level of TSP1’s known interaction with TGFβ, 2 month old Id cDKO mice were given LSKL (Anaspec), a known peptide inhibitor of TSP1 that selectively disrupts TSP1-dependent TGFβ activation^12–15^. RT PCR analysis revealed that LSKL injected Id cDKO hearts demonstrated a 3.61 ± 0.19 (p < 0.05) reduction in Col1A1 expression compared to non-LSKL injected controls (Fig. 6A). mRNA expression of TGFβ2, TGFβ3 and Col3A1 were not significantly altered. Western blot analysis for Col1A1 revealed a significant 20% reduction in Col1A1 protein level in LSKL injected Id cDKO mice compared to non-LSKL injected control levels (Fig. 6B). These trends were tested *ex vivo* using a cardiac explant culture system where 1 mm transverse cardiac sections from WT, Id control and Id cDKO mice were incubated in 20 μM LSKL at 37 °C for 6 hrs with 5% CO_2_ under rocking conditions. RNA was subsequently extracted and analyzed via real-time quantitative PCR. CTGF mRNA expression showed a significant 2.62 ± 0.53 fold reduction (p < 0.05) in Id cDKO treated with LSKL vs. untreated controls. Col3A1 mRNA expression showed a significant 2.15 ± 0.18 fold reduction (p < 0.05) in Id cDKO treated with LSKL vs. untreated controls. A trend towards decreased expression of Col1A1 was observed in Id cDKO treated with LSKL vs. untreated control but was not statistically significant (Fig. 6C).

## Discussion

While Id double knockout mice die at midgestation with numerous cardiac defects, prior single Id knockout studies have not yet reported any significant cardiac phenotypes. This observation coupled with the finding that Id1 and Id3 are expressed in overlapping patterns within the embryo^16^ suggest that dual Id1 and Id3 deletion accomplished through the Tie2Cre/loxP system effectively eliminated Id compensation in a layer specific manner (endothelium, endocardium). These mice survived into adulthood and developed a distinct fibrotic cardiomyopathy phenotype characterized by endocardial and endomyocardial disruption and fibrosis, reduced cardiac function, hypertrophic changes and increased perivascular fibrosis^4^. Aged 12–14 month old Id1 knockout mice lacking three alleles of Id (Id1^−/−^Id3^+/−^) do not exhibit signs of cardiac pathology. Conversely, aged 12–14 month old Id control mice (Id1^F/−^Id3^−/−^) develop signs of cardiac disease characterized by endocardial fibrosis reminiscent of early Id cDKO pathology, suggesting that Tie2Cre mediated ablation of Id compensation results in acceleration of cardiac pathology as Id cDKO hearts typically present with fulminant pathology by 6 months of age. The observation of mice with only a single functional allele of Id3 do not exhibit evidence of cardiac disease while mice with only a single functional allele of Id1 do exhibit early signs of cardiac disease at an advanced aged suggests that while Id1 and Id3 may compensate, the degree to which the two Id members functionally compensate may differ.

Through this study, Id cDKO hearts were shown to overexpress TSP1 with a pattern of distribution that appears interstitial, localizing to endothelial cells with increased concentration in the endocardial region near regions of disruption. In light of prior evidence that Id1 represses TSP1 transcription to mediate the process of angiogenesis^8^, these findings coupled with the observation of decreased CD31 density within the endocardium suggest that loss of Id1 repression leads to increased TSP1 expression may in turn play a role in defective vascularization of Id cDKO hearts. Prior reports have demonstrated that TSP1 mediates apoptosis through activation of the Bax/Bcl-2/caspase 3 pathway in endothelial cells^10, 17, 18^. Our studies reveal increased levels of apoptosis in Id cDKO hearts. Whether this observed pattern of increased apoptosis is endothelial remains to be determined.

Reports of TSP1’s role in cardiac fibrosis remain controversial and highly context dependent^14, 19–23^. In this system of Id loss, TSP1 appears to contribute in part to the fibrotic phenotype observed through TSP1-dependent activation of TGFβ and downstream upregulation of CTGF and collagens. Direct antagonism of this interaction between TSP1 and TGFβ using LSKL (an inhibitory peptide derived from latency associated peptide) appears to block this cascade with evidence of reversal of CTGF and collagen expression patterns. Whether a similar effect can be achieved *in vivo* through selective agents targeting downstream members of this cascade remains to be determined.

To address the fact that Tie2 targets both hematopoietic and endothelial cell lineages and determine whether the hematopoietic defects observed in Id cDKOs contribute to the observed cardiomyopathy, a series of bone marrow transplantation experiments were performed. Roughly 62% of RBMTs demonstrated evidence of incipient cardiac pathology reminiscent of the Id cDKO cardiac pathology. Cardiac function was not compromised at 4 months post-transplantation. Future long-term follow-up studies will help determine whether cardiac pathology becomes evident and cardiac function declines over time. The bone marrow transplantation results suggest that the absence of Id signaling in the blood can partially trigger fibrosis in endomyocardial regions of a WT heart. The effect of RBMT in the heart, however, is milder compared to that of the Tie2CRE Id cDKO mice. The mild fibrotic phenotype could be due to the fact that the hematopoietic but not the endothelial lineage is Id-defective in the RBMT, and also due to the fact that the switch from Id positive to Id negative in RBMT takes place at 2 months, while in Id cDKO mice it takes place well before during mid-gestation development. Increased expression of TSP1, CTGF, Col1A1 and Col3A1 suggest that TSP1/TGFβ/CTGF/collagen are activated in RBMT hearts. Whether injection of agents like LSKL that disrupt this signaling cascade into RBMT mice can help prevent the emergence of cardiac pathology remains to be determined. While anemia may contribute to the cardiac disease over time, RBMT mice develop evidence of cardiac disease in the absence of anemia, suggesting that anemia in this system may not contribute significantly to the development of this cardiac phenotype.

We have previously described a non-cell autonomous role for the mode of action of Id genes, in particular in the developing heart. The Id genes govern the expression of short- and long-range secreted factors, including Wnt5a and IGF-1 respectively^3, 24^. In the absence of Id1 and Id3 genes, IGF-1 is downregulated in the developing epicardium, which directly affects the proliferation rate of the developing myocardium at mid-gestation. Intraperitoneal injection of IGF-1 into mothers harboring Id1Id3 KO embryos partially rescues the lethal cardiac phenotype. In this report, we established a link between loss of Id in the hematopoietic system and upregulation of TSP-1, a known target of Id genes^8^, in the myocardium, with direct consequences in endomyocardial pathology and downstream fibrotic factors. Whether the soluble factor that bridges Id loss distally and upregulation of TSP is IGF-1, it remains to be further elucidated. These findings may have significant clinical implications for screening of bone marrow donors in the assessment of long-term safety of bone marrow transplantation therapies.

## Electronic supplementary material

Supplementary Info
