# Supplementary material for: Hematopoietic Id Deletion Triggers Endomyocardial Fibrotic and Vascular Defects in the Adult Heart
Source: Sci Rep. 2017 Jun 8;7:3079. doi: 10.1038/s41598-017-03160-7 (PMC5465087; doi:10.1038/s41598-017-03160-7)
Supplement: Supplementary file 1 — Supplementary Info [file 41598_2017_3160_MOESM1_ESM.pdf]

Hematopoietic Id Deletion Triggers Endomyocardial Fibrotic and Vascular Defects in the Adult Heart

Corey Chang[#], Qingshi Zhao[#], J. Patrick Gonzalez, Jung H. Kim, Kamal Alzahrani, Dominic Del Re, Diego Fraidenraich*

Department of Cell Biology and Molecular Medicine, New Jersey Medical School, Rutgers Biomedical and Health Sciences, 185 South Orange Avenue/Medical Science Building G-624, Newark, NJ 07103-2501 United States of America

Short Title: Hematopoietic Id loss leads to cardiac defects

To whom correspondence should be addressed: Diego Fraidenraich PhD, Department of Cell Biology and Molecular Medicine, New Jersey Medical School, Rutgers Biomedical and Health Sciences, 185 South Orange Ave, MSB G609, Newark, NJ 07103-2501, USA, Tel: (973) 972-5789; E-mail: fraidedi@rutgers.edu

* Corresponding author

E-mail: fraidedi@rutgers.edu

[#] Equal contribution

Keywords: Cardiac fibrosis, thrombospondin-1, bone marrow, endothelial cells, Id1, Id3

SUPPLEMENTARY INFORMATION

METHODS

Serum Isolation/Preparation

Whole blood was collected from the retroorbital isnus into untreated 1.5 mL eppendorf tubes and allowed to sit at room temperature for 1 hr. Samples were then centrifuged at 3000 rpm (1500 x g) for 15 mins in 4°C. The serum was carefully removed from the top leaving behind the interphase buggy coat and red blood cells. Serum was aliquoted and stored in -80°C.

Western Blot Analysis

Snap frozen left ventricular tissue was immediately suspended in RIPA buffer (containing 150 nM sodium chloride, 1.0% NP-40, 0.5% sodium deoxycholate, 0.5% SDS, 50 nM Tris pH 8.0) and supplemented with PhosStop phosphatase inhibitor (Roche) and cOmplete Ultra protease inhibitor cocktail (Roche). Samples were homogenized over ice and spun down at 12000 rpm in 4°C to remove insoluble debris. Samples were loaded with 4X Laemmli buffer (BioRad) and protease inhibitor (Sigma) and heated at 99°C for 10 mins before loading onto 4-20% SDS gradient gels. Gels were run at 120 mV and transferred onto nitrocellulose at 75 mV in 4°C for 2 hrs. Nitrocellulose membranes were stained with Ponceau S stain to check for even loading prior to antibody probing.

Real Time qPCR

Snap frozen tissue samples were immediately suspended in Trizol. Total RNA was extracted according to manufacturer's protocol and 1 µg of total RNA was reverse transcribed using Superscript III reverse transcriptase (Invitrogen).

Primers were designed from mouse sequences corresponding to the genes of interest provided below:

Primer	Sequence
β-actin	s-5'-AGCCATGTACGTAGCCATCC-3'
	as-5'-CTCTCAGCTGTGGTGGTGAA-3'
Tsp1	s-5'-GTCACATCGCCAACACAATC-3'
	as-5'-AGACCAAAGCCTGCAAGAAA-3'
TGFβ1	s-5'-CAACAATTCCTGGCGTTACCTTGG-3'
	as-5'-GAAAGCCCTGTATTCCGTCTCCTT-3'
Ang1	s-5'-TCTGCACAGTCTCGAAATGG-3'
	as-5'-AGGCTTGGTTTCTCGTCAGA-3'
Ang2	s-5'-ACTACGACGACTCAGTGCAA-3'
	as-5'-TTTGTGCTGCTGTCTGGTTC-3'
CTGF	s-5'-CAAAGCAGCTGCTGCAAATACCA-3'
	as-5'-GGCCAAATGTGTCTTCCAGT-3'
Col1a1	s-5'-CCTGCCTGCTTCGTGTAAACT-3'
	as-5'-TTGGGTTGTTCGTCTGTTTCC-3'
Col3a1	s-5'-TTGATGTGCAGCTGGCATTC-3'
	as-5'-GCCACTGGCCTGATCCATAT-3'
Id1	s-5'-TCCTGCAGCATGTAATCGAC-3'
	as-5'-TTTTCCTCTTGCCTCCTGAA-3'
Id3	s-5'-ACTCAGCTTAGCCAGGTGGA-3'
	as-5'-GTCAGTGGCAAAAGCTCCTC-3'

After reverse transcription (RT), the cDNA was used for quantitative PCR (40 cycles of a 10 s-step at 95°C and a 1 min-step at 60°C) with SybrGreen (Applied Biosystems) on a 7300 Sequence Detector (Applied Biosystems). Fold change was calculated using the $2^{-\Delta\Delta Ct}$ method. Values are normalized against β-actin, a housekeeping gene

Histology

Tissues were fixed in 4% PFA overnight at 4°C and were subsequently placed in 70% ethanol prior to submission to the Histology Core Facility for processing and paraffin embedding. Coronal sections were mounted onto Superfrost glass slides (ThermoScientific) using a microtome and placed in a 55°C oven for 10 mins before beginning the staining process to anneal the specimens to the slide. Sections were subsequently deparaffinized and hydrated prior to staining.

For H&E stain, sections were then stained using hematoxylin (Gill's; VectorLabs) and 95% eosin as per manufacturer's protocol. Masson Trichrome Stain was performed using the Masson Trichrome Stain kit following manufacturer's protocols (Richard-Allan Scientific). Images were acquired using a Nikon Eclipse 80i microscope with NIS Elements AR software.

To quantify endomyocardial fibrosis, images were blindly analyzed for fibrosis using ImageJ software. A grid mask was applied to each image to delineate equally sized regions of 0.104 mm^2. In five randomly selected fields within endomyocardial regions of the left ventricle (1/3 inner area of the myocardium), areas of fibrosis, muscle and empty space were identified and

given different pseudo-colors (blue for fibrosis, red for muscle). Once all of the histological features were identified, data were exported directly to Microsoft Excel and the number of regions containing endomyocardial fibrosis was normalized by the number of regions containing muscle as follows: (blue)/(red) x 100, which represents (number of regions containing fibrosis)/(number of regions containing cardiac muscle) x 100. WTBMT: n = 2 and RBMT: n = 4. n = number of mice. All coronal sections were obtained at mid-ventricular level.

To quantify collagen content, hydroxyproline content was determined with 10 mg of homogenized WTBMT and RBMT cardiac tissue using the hydroxyproline Assay Kit (Sigma-Aldrich), according to the manufacturer's recommendation.

Immunofluorescence/Immunohistochemistry

Myocyte Cross Sectional Area Analysis

Following deparaffinization and hydration to ddH_2O and 1X PBS, sections were incubated for 30 mins with wheat germ agglutinin conjugated with Alexa Fluor 546 (red) diluted at 1:1000 in Hank's Buffered Saline Solution (HBSS) and washed in two changes of 1X PBS prior to mounting with Vectashield Mounting Medium with DAPI counterstain (Vector). Images were acquired using a Nikon Eclipse 80i microscope with NIS Elements AR software. Images were analyzed for myocyte cross sectional area using ImageJ software. Measurements were obtained from five randomly selected fields within the endocardial region of the heart.

TSP1 Immunohistochemistry

Following deparaffinization and hydration to ddH_2O, sections were immersed in citrate buffer pH 6.0 (Thermo Scientific) and microwaved for 1.5 mins and subsequently placed in the pressure cooker for 20 mins followed by cooling at 20 mins at room temperature. Elements of the LP system kit were used for this stain (Thermo Scientific). Briefly, sections were immersed in hydrogen peroxide block for 15 mins to reduce nonspecific background staining from endogenous peroxidase activity. Ultra V block was then applied to the sections for 5 mins at room temperature after which point the TSP1 Ab-4 (clone A6.1) monoclonal mouse anti-thrombospondin-1 antibody (NeoMarkers) was applied for 1 hr at room temperature at a dilution of 1:1000 in 1X PBS. Sections were then incubated in primary antibody enhancer for 20 mins, HRP polymer for 30 mins and AEC single solution for 10 mins with four 1X PBS washes in between each step. HRP polymer and AEC single solution steps were performed in the dark. Slides were then counterstained with hematoxylin stain (Gill's formula, Vector) for 2 mins followed by a water wash and incubation in 0.08% NH_4OH solution and aqueous eosin (Sigma) for 1 min. Aqueous eosin was made up as 0.5 mL of acetic acid per 100mL of aqueous eosin. Sections were then rinsed with tap water and mounted with Shur/Mount aqueous liquid mounting medium (TBS).

CD31 Quantification

Following deparaffinization and hydration to ddH_2O, sections were immersed in citrate buffer pH 6.0 (Thermo Scientific) and microwaved for 1.5

mins and subsequently placed in the pressure cooker for 20 mins followed by cooling at 20 mins at room temperature. Sections were then treated with 3% hydrogen peroxide for 10 mins at room temperature to quench endogenous peroxidase activity followed by permeabilization with 0.3% Triton X-100 in 0.05% PBST for 10 mins (Fisher Scientific). Sections were subsequently blocked with DAKO serum-free blocking buffer (DAKO) for 60 mins and incubated in rabbit polyclonal anti-CD31 antibody (Abcam) at a dilution of 1:100 in DAKO antibody diluent with background reducing components (DAKO) overnight in 4°C. Sections were then incubated with Alexa Fluor 555 donkey anti-rabbit antibody at a dilution of 1:250 in DAKO antibody diluent for 60 mins at room temperature after which sections were washed in 1X PBST and mounted with Invitrogen Antifade Gold Reagent with DAPI (Invitrogen). Slides were imaged using a Nikon Eclipse 80i microscope and analyzed with grid overlay using the ImageJ64 software. Five randomly selected fields in the endocardium were selected for CD31 quantification. Results are tabulated as CD31 positive cells per field.

TUNEL Assay

Following deparaffinization and hydration to ddH_2O and 1X PBS, sections were treated with 20 µg/mL of proteinase K in 1X PBS for 30 mins at room temperature. Sections were then washed and incubated in TDT (terminal deoxynucleotidyl transferase) reaction mixture for 60 mins at 37°C. TDT reaction mixture consists of 40 µL TDT reaction buffer, 140 µL milli-Q water, 20 µL $CoCl_2$ solution, 20 units of TDT and 2 nM biotin-16-dUTP. Sections were mounted with Shur/Mount aqueous liquid mounting medium (TBS). Sections were imaged

using the Nikon Eclipse 80i microscope and NIS Elements AR software. Images were analyzed using IPWIN32 image quantification software and reported as percentage of positive cells per DAPI per field.

TSP1/CD31 Dual Immunostain and Confocal Microscopy

Following deparaffinization and hydration to ddH_2O and 1X PBS, sections were immersed in citrate buffer pH 6.0 (Thermo Scientific) and placed in the pressure cooker for 20 mins followed by cooling at room temperature for 20 mins. Sections were then treated with 3% hydrogen peroxide (Fisher Scientific) for 10 mins to quench endogenous peroxidase activity. Permeabilization was achieved by incubating sections in 0.3% Triton X-100 in 1X PBST for 10 mins. Streptavidin/biotin blocking was performed using the Streptavidin/Biotin Blocking Kit (Vector) as per manufacturer's protocol. Sections were then stained for CD31 as detailed in the 'CD31 Quantification' section of the Methods section. TSP1 co-stain was performed using the Vector M.O.M. (mouse on mouse) Immunodetection Kit (Vector). Prior to TSP1 staining, sections were blocked with Mouse Ig Blocking Reagent for 1 hr at room temperature. Tissue sections were then incubated with TSP1 Ab-4 (clone A6.1) monoclonal mouse anti-TSP1 antibody (NeoMarkers) in working solution of M.O.M. Diluent (prepared by combining Protein Concentration stock solution with 1X PBS at a 1:12.5 dilution) at an antibody dilution of 1:1000 at room temperature for 1 hr. Biotinylated anti-mouse IgG reagent in M.O.M. diluent was then applied for 10 mins at room temperature, followed by incubation with ABC reagent (Vector) and Streptavidin Alexa Fluor 488 (green) at a dilution of 1:250 in 1X PBS for 1 hr in room

temperature in the dark. Sections were then mounted with Invitrogen Antifade Gold with DAPI counterstain. Images were acquired via confocal microscopy using the Nikon A1R on Nikon Eclipse TI inverted base.

GFP/CD31 Dual Immunostain

Following deparaffinization and hydration to ddH_2O and 1X PBS, sections were immersed in citrate buffer pH 6.0 (Thermo Scientific) and placed in the pressure cooker for 20 mins followed by cooling at room temperature for 20 mins. Sections were then treated with 3% hydrogen peroxide (Fisher Scientific) for 10 mins to quench endogenous peroxidase activity. Permeabilization was achieved by incubating sections in 0.3% Triton X-100 in 1X PBST for 10 mins. Sections were then stained for CD31 at dilution of 1:100 as detailed in the 'CD31 Quantification' section of the Methods section. Prior to GFP staining, sections were incubated in DAKO serum-free blocking reagent (DAKO) for 1 hr at room temperature. Sections were then incubated in chicken polyclonal anti-GFP antibody (Abcam) at a dilution of 1:500 in DAKO Antibody Diluent with background reducing components for 1 hr at room temperature followed by incubation with Dylight 488 goat anti-chicken IgG antibody at a dilution of 1:250 in DAKO Antibody Diluent. Sections were subsequently mounted with Vectashield Mounting Medium with DAPI (Vector) and imaged using the Nikon Eclipse 80i and NIS Elements AR software.

Echocardiography

Echocardiographs were performed on WT, Id control, Id1 KO, Id cDKO, WTBMT, FBMT and RBMT mice to assess cardiac function. Mice were

anesthetized via intraperitoneal injection with 2.5% Avertin at a dosage of 290 mg/kg. Transthoracic echocardiography was performed using a 13 MHz linear ultrasound transducer (VisualSonics Vevo 770 High Resolution in Vivo Micro Imaging System). After shaving the chest, mice were placed on a flat rotational platform under a warming lamp. The limbs were taped to electrodes to record a live EKG and a rectal probe was inserted to monitor fluctuations in body temperature during the procedure. Mice were oriented in a shallow left lateral position and warm coupling gel was applied to the chest. Two-dimensional images and left ventricular M-mode tracing were acquired from the parasternal short axis view at a sweeping speed of 100-200mm/s at the level of the mid-papillary muscle. M-mode measurements of left ventricular internal diameter (LVID) and wall thickness were acquired over 3 consecutive beats and averaged using the leading edge-to-leading edge convention adopted by the American Society for Echocardiography. Heart rate was maintained between 400-600 beats per minute. End-diastolic measurements were acquired at the peak of the R wave on EKG recordings. End-systolic measurements were acquired at the most anterior systolic excursion of the posterior wall. Left ventricular ejection fraction (LVEF) was calculated following the cubed method using the following formula: LVEF (%) = 100 x $[(LVIDd)^3-(LVIDs)^3] / (LVIDd)^3$ where LVIDd is the left ventricular end diastolic dimension and LVIDs is the left ventricular and systolic dimension. Fractional shortening (FS) was calculated as: FS (%) = 100 x [(LVIDd-LVIDs) / LVIDd]

Supplementary Figure 1

Uncropped image of blot represented in Figure 5C. Top: TSP1. Bottom: GAPDH

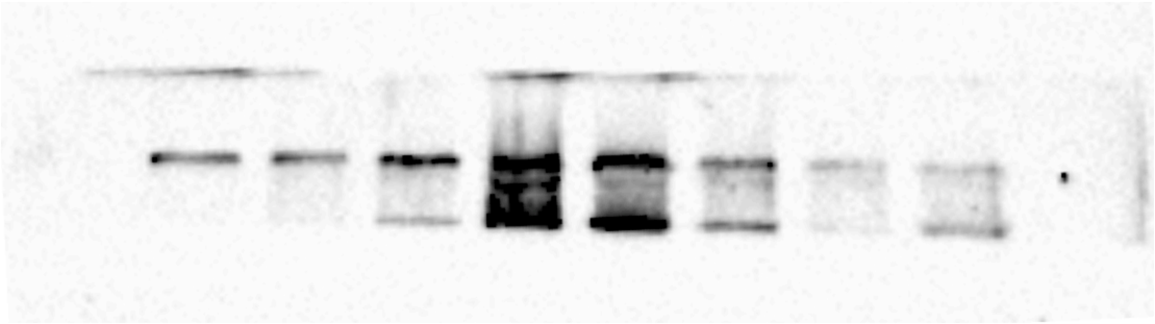

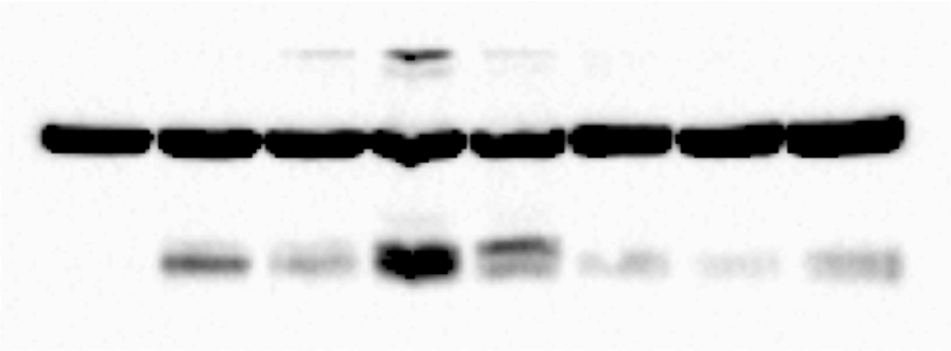

Supplementary Table 1. Summary of histological analysis of bone marrow transplanted mice. The top table summarizes the histological findings in the hearts of RBMT mice. The bottom table summarizes the histological findings in the hearts of FBMT mice.

Histological Analysis of RBMT Hearts

Donor	Recipient	Evidence of Endocardial Fibrosis?	Disruption of Endocardial Lining?	Ratio of Recipients with Phenotypic Evidence of Cardiac Pathology
#68 cDKO	#1 WT	Yes	Yes	
	#12 WT	No	No	1/2
#53 cDKO	#3 WT	Yes	Yes	
	#4 WT	Yes	Yes	2/2
#19 cDKO	#6 WT	No	Yes	1/1
#140 cDKO	#10 WT	No	No	
	#11 WT	No	No	0/2
#175 cDKO	#3 WT	Yes	Yes	
	#4 WT	No	No	
	#5 WT	Yes	Yes	2/3
#35 cDKO	#1 WT	Yes	Yes	
	#2 WT	Yes	Yes	
	#3 WT	No	No	2/3

Histological Analysis of FBMT Hearts

Donor	Recipient	Evidence of Endocardial Fibrosis?	Disruption of Endocardial Lining?	Ratio of Recipients with Phenotypic Evidence of Cardiac Pathology
#1 WT	#110 cDKO	Yes	Yes	
	#122 cDKO	No	No	1/2
#2 WT	#44 cDKO	Yes	Yes	
	#176 cDKO	Yes	Yes	
	#39 cDKO	No	No	2/3
#3 WT	#58 cDKO	Yes	Yes	
	#60 cDKO	Yes	Yes	
	#63 cDKO	Yes	Yes	3/3
#4 WT	#64 cDKO	No	No	
	#66 cDKO	Yes	Yes	1/2
